# Editorial: Long-term durability of biological aortic valves

**DOI:** 10.3389/fcvm.2024.1381366

**Published:** 2024-03-04

**Authors:** Thierry Caus, Bart Meuris, Jean François Avierinos

**Affiliations:** ^1^ Department of Cardiac Surgery, University Hospital Amiens-Picardy, Amiens, France; ^2^ Department of Cardiac Surgery, Universitair Ziekenhuis, Leuven, Belgium; ^3^ Department of Cardiology, University Hospital Timone, Marseille, France

**Keywords:** biological valves, mechanical valves, imaging, calcification, VARC III

**Editorial on the Research Topic** Long-term durability of biological aortic valves

With over 6,000 total views and downloads to date, this short, but contributive article collection is dedicated to the still unsolved problem of Long Term Durability of Biological Aortic Valves. Each co-editor of this research topic contributed efficiently by writing, mentoring or editing high quality manuscripts, which underwent, thereafter, an independent and strict reviewing process. We thankfully transmit our respects to all authors but also to those very engaged reviewers who entirely contributed to enhance the quality of the papers, which were eventually published (see Table 1). Unfortunately, this process also discarded some other valuable contributions, which will surely find some opportunities to be published elsewhere.

**Table 1:** List of articles accepted in the Research Topic.

Authors	Title	Doi	Main finding
Chabry et al.	Prevention by the CXCR2 antagonist SCH527123 of the calcification of porcine heart valve cusps implanted subcutaneously in rats	https://doi.org/10.3389/fcvm.2023.1227589	To inhibit the action of IL-8 by blocking its CXCR2 receptor prevents calcifications of heart valve cusps in a xenogeneic allograft in-vivo model
Porto et al.	One-year clinical outcomes following Edwards INSPIRIS RESILIA aortic valve implantation in 487 young patients with severe aortic stenosis: a single-center experience	https://doi.org/10.3389/fcvm.2023.1196447	This new generation of decellularized tissue valve gives promising early clinical results. Adoption of standardized VARC-3 outcome report will help to better define late results
Vermes et al.	Is there a role for cardiovascular magnetic resonance imaging in the assessment of biological aortic valves?	https://doi.org/10.3389/fcvm.2023.1250576	Cardiovascular magnetic resonance imaging is mainly a help to existing diagnosis tools to detect tissue valve failure. CMR is therefore unlikely to initiate a VARC-4 outcome report
Caus et al.	Trends in SAVR with biological vs. mechanical valves in middle-aged patients: results from a French large multi-centric survey	https://doi.org/10.3389/fcvm.2023.1205770	The authors relate on a recent shift from mechanical to biological SAVR in middle-aged patients based on a nation-scale study in France. This shift is contemporary from the results of PARTNER II and is likely to persist with the exponential current use of TAVR in young and low-risk patients
Carrel et al.	Evolving technology: the TRIFLO tri-leaflet mechanical valve without oral anticoagulation: a potential major innovation in valve surgery	https://doi.org/10.3389/fcvm.2023.1220633	The authors announce a revolution to come in the world of mechanical heart valves, which has been marked for decades by the lack of true technical evolutions. By avoiding long-term anticoagulant therapy, this could annihilate the need for a biological valve more durable than current ones

Neither optimistic nor pessimistic about the studied question, the editorial line of the current research topic aims at being realistic and factual. Reflecting its broad spectrum, this paper collection includes one brief research report, two original research articles as well as two reviews.

Tissue valves, though biological by nature, are not utterly biocompatible and long-term durability is impaired by fatigue lesions as well as by calcifications, which are the ultimate consequences of immunological and inflammatory processes not so far from tissular rejection. By elegantly studying *in vivo*, the effects of antagonists of chemokine receptor type 2 on valvular calcifications, Chabry et al. stress on the importance of the inflammatory response to the durability of tissue valvular and suggest a potential preventive treatment of SVD in high risk patients. Though this approach is still very preliminary, we believe that it might prove to be interesting in future.

During the past few years, the common knowledge about the durability of biological valves has been challenged by the new VARC-3 consensus about the definition of tissue primary failure. Based on that, surgical series have to be updated to this new paradigm and to report their results accordingly. Adopting the VARC-3 definition consensus, Porto et al. report on the largest ever-published series of patients implanted with the new Edwards INSPIRIS RESILIA, a tissue valve device that aims at greatly improving biocompatibility of tissue valves. Though the follow-up duration is still limited, results are excellent and we eagerly await data from long term follow-up.

Because the definition of biological valve failure has evolved with the accuracy to detect tissular abnormalities with imaging techniques, VARC-3 now defines a Stage 1 structural valve deterioration (SVD) as morphological valve deterioration without significant hemodynamic changes. To investigate the potential role of cardiac magnetic resonance (CMR) imaging on the assessment of biological aortic valves, Vermes et al. performed a well-illustrated review, which demonstrates the value of CMR as a complementary tool when the results of valve evaluation by trans-thoracic or trans-esophageal echocardiography are equivocal or inconclusive for any reason.

Best practice guidelines, which aim at adhere closely to scientific evidences often end up to resume being best wishes when confronted to the market-driven real world practices. By studying tendencies in the implantation of biological or mechanical SAVR across a recent period in France, Caus et al. demonstrate an obvious shift towards biological SAVR in middle-aged patients and question the rationale for it with regards to the literature supporting guidelines. This situation is unlikely to reverse soon, if Western Europe is to follow trends in USA, where TAVI procedures are currently broadly performed in patients under 65-years-old.

We might be just one leaflet away from the ideal valve substitute, which would offers both biocompatibility and durability. Just before performing the first in man, Carrel et al. propose a comprehensive review of the TRIFLO valve and underline the importance of the reverse flow phase of current bi-leaflet mechanical valves in platelet activation and thrombosis formation. Therefore, it is more this peculiar hemodynamic characteristics, shared by all current mechanical prosthetic valves, than the nature of foreign material used to build them, which makes the need for permanent oral anticoagulation. Ultimately, optimizing the design of mechanical valves and adopting a tri-leaflet configuration could lead to obtain a cheap, universal, biocompatible and reliable valvular substitute to treat all forms of surgical valve disease worldwide.

We encourage the lectors of this editorial to take a look at all articles related to the current research topic of Long Term Durability of Biological Aortic Valves. We wish them to get during lecture as much pleasure as we have experienced when elaborating this collaborative work under the flag of Frontiers!

